# Iatrogenic seeding of osteosarcoma in a tracheotomy tract – a case report

**DOI:** 10.1002/ccr3.1395

**Published:** 2018-01-26

**Authors:** Irit Duek, Richard Lopchinsky, Sharon Akrish, Ziv Gil

**Affiliations:** ^1^ Department of Otolaryngology Head and Neck Surgery The Head and Neck Center Rambam Health Care Campus Affiliated to the Rappaport Institute of Medicine and Research The Technion ‐ Israel Institute of Technology Haifa Israel; ^2^ Department of Pathology The Head and Neck Center Rambam Health Care Campus Affiliated to the Rappaport Institute of Medicine and Research The Technion ‐ Israel Institute of Technology Haifa Israel

**Keywords:** Head and neck surgery, iatrogenic seeding, osteosarcoma, tracheotomy tract seeding

## Abstract

Here, we describe the first reported case of osteosarcoma occurring postsurgically in a tracheotomy tract, highlighting the possibility of osteosarcoma seeding during head and neck surgery. Preventative measures such as performing the tracheostomy after the tumor resection while walling off the tracheostomy site from the operative field should be considered.

## Introduction

Osteosarcoma is the most common primary malignant bone tumor in children and adolescents. However, it rarely occurs in the head and neck region, accounting for less than 1% of head and neck malignancies 1. The vast majority occur in the mandible and maxilla 2. The primary presenting complaints are pain, swelling, paresthesia, and ulceration 3. Head and neck osteosarcomas have distinct biological and clinical features that distinguish them from osteosarcomas in the extremities. In the head and neck, they usually affect older people with the median age in the fourth decade 2. They are more likely to recur locally after treatment, and distant metastases are observed less often than with the more common osteosarcomas arising in the long bones 2. Although the combination treatment of neoadjuvant chemotherapy and surgery has greatly improved the oncological outcome of extremity osteosarcoma 1, the optimal therapeutic strategy for head and neck osteosarcoma is still to be defined. The p53 mutation is associated with an aggressive behavior in osteosarcoma and with multifocal osteosarcoma. Park et al. 4 found that the presence of p53 oncogene in osteosarcoma was associated with a shorter survival, increased tumor activity, and drug resistance. In osteosarcoma, local recurrence is a major concern after biopsy or definitive surgery. Removing the primary tumor can have a permissive effect on distant metastases. Implantation of circulating or dormant cancer cells in sites of tissue injury and wounds can be stimulated by trauma or inflammation. This mechanism is one of several postulated to account for local recurrences. Other mechanisms include regrowth of tumor at positive margin or in a field of adjacent condemned mucosa. Intraoperative implantation of cancer cells should be guarded against in order to decrease local‐regional recurrence. A few reports indicate that tumor cell contamination and local metastasis can occur along the needle puncture track, in extremity osteosarcoma 5. It has been shown that after open biopsy, there is a much higher recurrence rate when the biopsy site has not been excised at definitive surgery (38% compared with 7%) 5. Here, we present the first case reported of iatrogenic seeding of osteosarcoma during a head and neck surgery, along a tracheotomy track.

## Case Presentation

**Figure 1:**
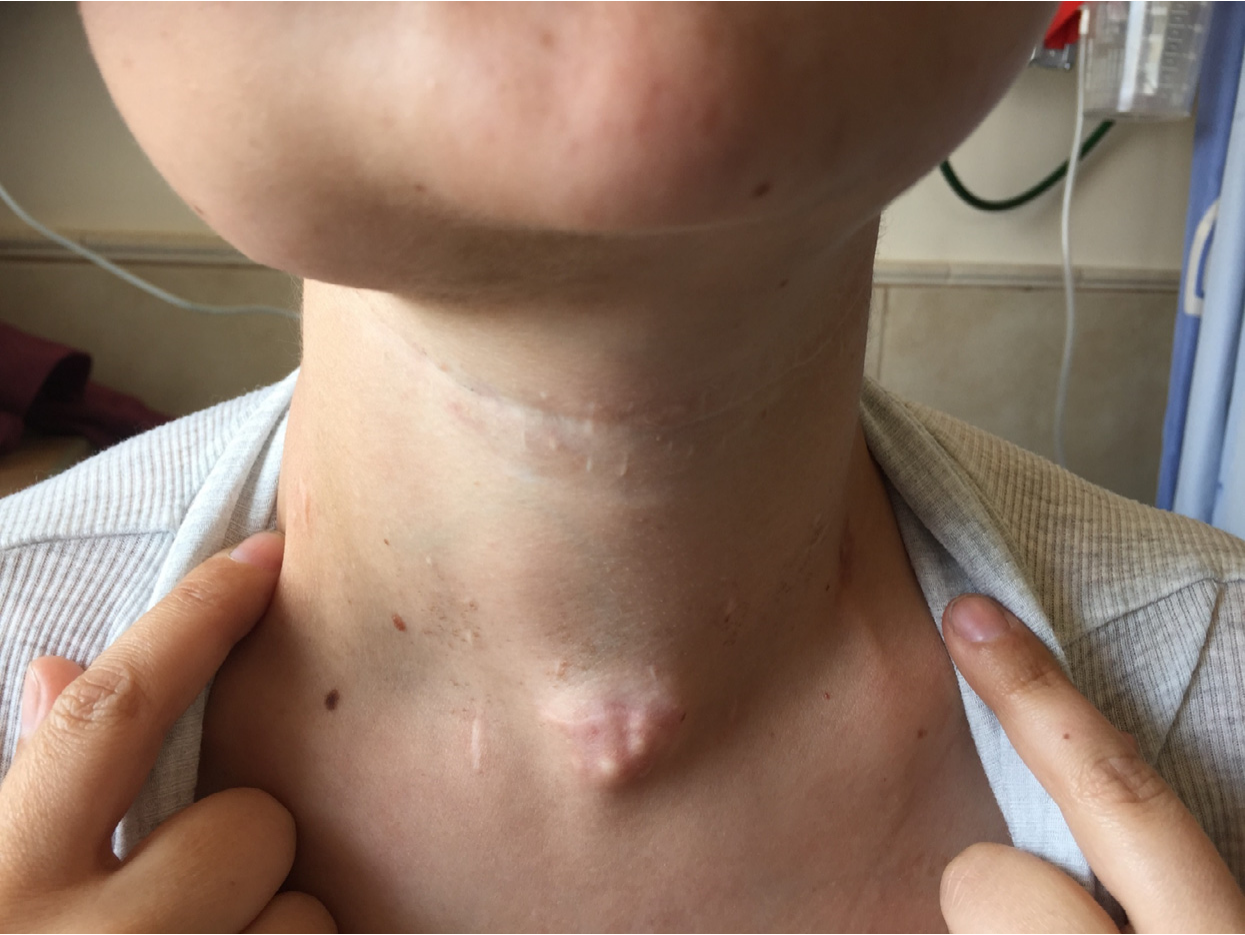
Preoperative picture of a 21‐year‐old patient with recurrent mandibular osteosarcoma, 6 months after composite resection and oncologic treatment. A lump at her central neck, at the scar of the tracheotomy incision, which was performed as a part of her previous operation, is noted.

**Figure 2:**
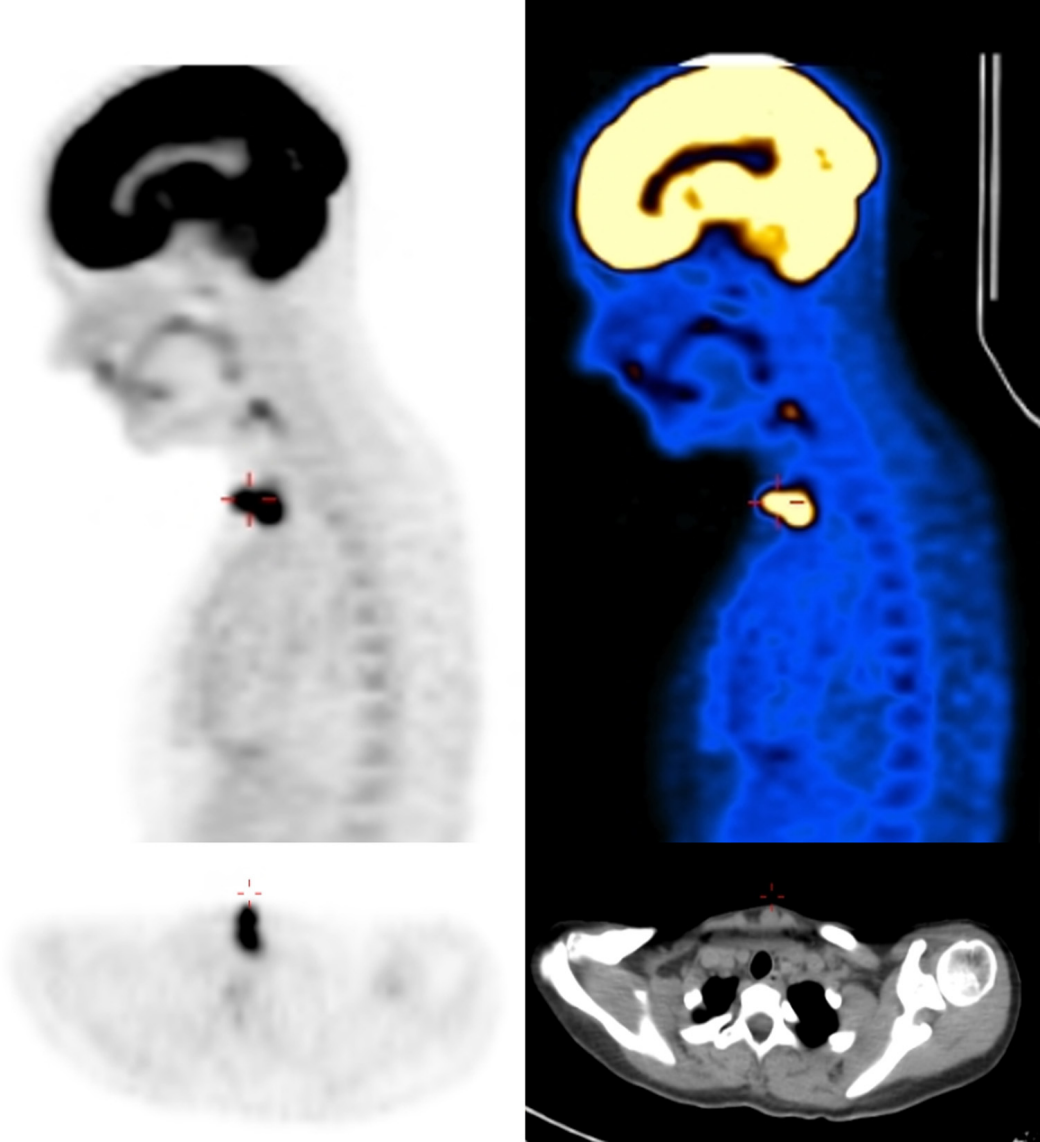
PET‐CT which was performed 6 months after the first mandibulectomy, and prior to last operation. A lump in the central neck, at the site of the tracheostomy scar, is noted. The lump shows FDG‐positive uptake.

**Figure 3:**
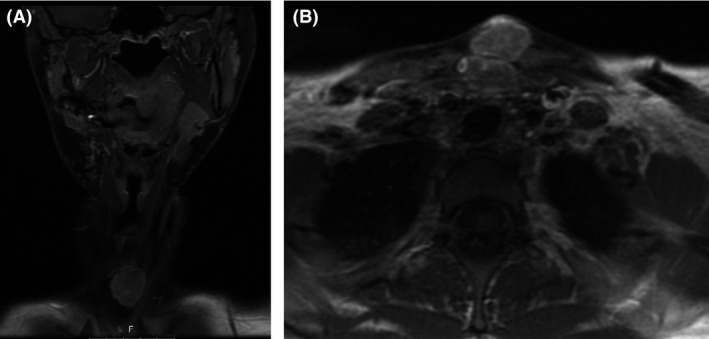
Preoperative coronal (A) and axial (B) gadolinium‐enhanced T1‐weighted MRI neck images of a 21‐year‐old patient with recurrent mandibular osteosarcoma, and iatrogenic osteosarcoma at the tracheotomy tract which was performed as a part of her previous operation.

**Figure 4:**
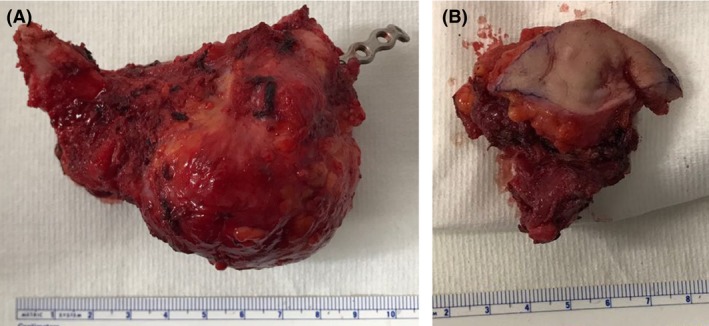
The specimens that were resected. The mandibular lesion (A) and the lumps from the tracheotomy tract (B) were resected completely.

**Figure 5:**
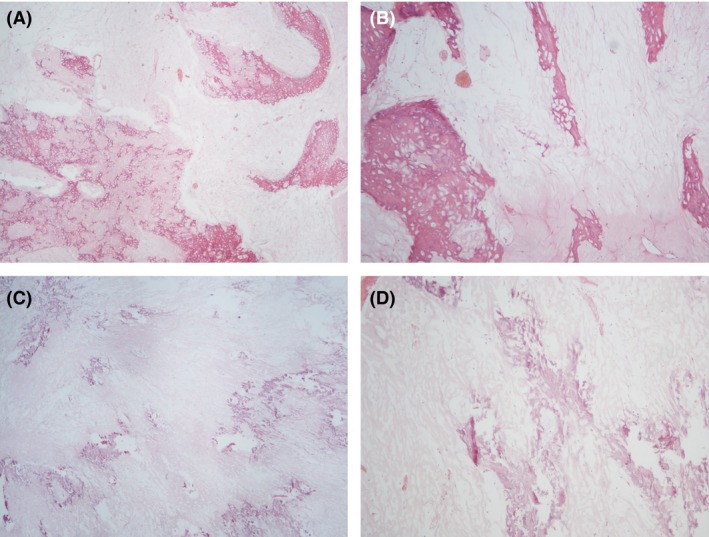
Microphotographs of the resected specimens’ histopathology sections. Hematoxylin and eosin staining; ×40 (A) ×100 (B) magnification of the mandibular lesion, compatible with osteosarcoma. The tumor mass is composed mostly of neoplastic mineralized bone in a fibrous tissue stroma. The paucity of tumor cells represents the effect of presurgical oncologic treatments. Hematoxylin and eosin staining; ×40 (C) ×100 (D) magnification of the tracheotomy tract lumps shows osteosarcoma cells that are subjacent to the skin epithelium, with no cartilage or skin invasion. No perineural invasion is seen.

A 21‐year‐old women was referred to our center due to recurrent mandibular osteosarcoma, 6 months after total tumor extirpation and postoperative radiation. She also was noted to have a lump at her central neck, at the scar of the tracheotomy incision, which was performed as a part of her last operation. The woman is a p53 mutation carrier, with a family history of a father who died from mandibular osteosarcoma. At 18 months of age, she was diagnosed with a right popliteal fossa rhabdomyosarcoma for which she underwent several surgeries and VACA‐IV chemotherapy for 18 months followed by radiation. At the age of 14 years, she was diagnosed with a postradiation osteosarcoma in her right tibia. She was treated with chemotherapy, followed by amputation with intercalary allograft implantation, a debridement after graft failure, and an anterior lateral thigh (ALT) free flap reconstruction (from her left leg). Further local osteosarcoma recurrences led to an above‐knee amputation of her right leg at the age of 15. At the age of 20, she noticed a gradually enlarging, symptomatic mass in her right mandible. A biopsy was taken, which revealed an osteoblastic osteosarcoma. At that time, she had a 3‐cm exophytic lesion in the right mandible, involving the floor of the mouth, extending under the mandible. The patient underwent a right segmental mandibulectomy, selective neck dissection levels 1‐3, and tracheotomy, with chimeric scapular and titanium plate flap reconstruction at another center. The histopathologic finding was a 13‐mm high‐grade osteosarcoma, involving the mandibular bone, gingival mucosa, and the masseter muscle. The anterior and posterior margins were free of tumor. One level 2 cervical lymph node, of 18 resected, showed metastatic tumor. Because of toxicities of her previous treatments with cisplatin, doxorubicin, etoposide (VP16), and ifosfamide, she received second‐line gemcitabine and docetaxel and eventually 2 cycles of methotrexate with good response according to the PET‐CT. Two months after surgery, treatment with pembrolizumab (Keytruda), an anti‐PD‐1 antibody, was initiated. However, swelling in her right mandible recurred. In addition, a lump in her central neck, at the site of the tracheostomy scar, was noted (Fig. 1) which showed FDG uptake in the PET‐CT that was performed 6 months after the surgery (Fig. 2). Fine‐needle aspiration (FNA) from this mass was consistent with osteosarcoma. At this point, 6 months after her operation, she presented at our center. A mobile firm nontender nodule was found on the central neck, at the site of the tracheotomy scar. Computerized tomography (CT) scan of the neck and magnetic resonance imaging (MRI) demonstrated 2 lumps, about 3 cm each, with the same characteristics as the mandibular lump, at the tracheotomy track (Fig. 3). She underwent a right mandibulectomy, with anterolateral thigh (ALT) free flap reconstruction, harvested from her right amputated leg. All the lesions, including the lumps from the tracheotomy tract, were resected completely with negative margins (Fig. 4). Histopathology revealed localized recurrences of osteosarcoma with clear resection margins. Tumor cells were found subjacent to the epithelium of the skin. The skin was not involved. There was no invasion to cartilage. No perineural invasion was seen. The minimal distance of tumor cells from surgical margins was 0.5 cm (Fig. 5). No intraoperative, perioperative, or postoperative complications were encountered. The patient was referred for local radiotherapy. At follow‐up, the patient was free of any symptoms, with no evidence of residual or recurrent disease in 12‐month postoperative MRI and PET‐CT.

## Discussion

Although malignant cancer cells seeding during oncologic surgery has been previously described, less attention has been given to the preventive measures and management strategies. Moreover, osteosarcoma seeding in a tracheotomy tract during head and neck surgery has not been previously described. Possible mechanisms for cancer cells seeding during surgery include direct contamination of surgical wounds with viable tumor cells and the spread of tumor cells in the blood circulation during manipulation of the primary lesion. Inflammation and trauma seem to stimulate the seeding of cancer cells at distant sites. The tracheotomy tract is prone for seeding via direct and hematogenous spread, and also via intraluminal contamination through aspiration of cancer cells 6.

In order to decrease the risk of malignant cells seeding during oncologic surgery, the following precautions measures can be taken: (1) Planning the operation carefully by first removing the primary lesion, and only after tumor removal, irrigation and site isolation, continuing toward and neck dissection and/or tracheotomy. (2) Meticulous irrigations with hypotonic saline solution after primary lesion removal. (3) Creating a mechanical barrier by covering the tracheotomy site and the primary lesion site, with a nonpenetrating wound dressing. (4) Changing gowns, gloves, and instruments after the primary lesion removal and before performing tracheotomy, reconstruction, and closure. (5) Consider including the entire operative field in the postoperative radiation field when postoperative radiation therapy is indicated.

## Conclusions

The case we present is not the usual seeding in the tract of a biopsy. To the best of our knowledge, this is the first case reported of iatrogenic seeding of head and neck osteosarcoma. Also, this is the first reported case of an osteosarcoma occurring in the tract of a tracheotomy that was carried out during mandibulectomy for mandibular osteosarcoma. This report highlights the possibility of osteosarcoma metastases occurring by direct implantation during head and neck surgery. Consequently, careful consideration should be given to possible preventative measures such as performing the tracheostomy after resection of the tumor and/or walling off the tracheostomy site from the operative field during resection of head and neck osteosarcoma.

## Authorship

ID: wrote manuscript, conception and design, participated in patient's management, operation, and follow‐up. RL: involved in drafting the manuscript, revising it critically for important intellectual content. SA: reviewed the pathological aspect of the case, examined the specimens, provided pathological diagnosis and histopathologic figures for the manuscript. ZG: managed the case, from first diagnosis and decision‐making, to planning and performing the operation and patient's follow‐up.

## Conflict of Interest

None of the authors has any conflicts of interest to disclose.
